# Conservation and Tandem Duplication of tRNA Genes in Plant Species

**DOI:** 10.3390/genes16111307

**Published:** 2025-11-01

**Authors:** Fan Zhang, Yajun Xiong, Yijie Chen, Sawaira Jadoon, Huan Yu, Zhiyu Liu, Kanglin Liu, Lijuan Qiu, Jun Wang

**Affiliations:** 1 The Shennong Laboratory, Zhengzhou 450002, China; haas_zhangfan@163.com (F.Z.);; 2 National Key Facility for Gene Resources and Genetic Improvement (NFCRI)/Institute of Crop Sciences, Chinese Academy of Agricultural Sciences, Beijing 100081, China; 3 Institute of Crop Molecular Breeding, Henan Academy of Agricultural Sciences, Zhengzhou 450002, China

**Keywords:** tRNA, conservation, tandem duplication, plant species

## Abstract

As an evolutionary ancient molecule, transfer RNA (tRNA) is ubiquitous across all domains of life as a living fossil. Background/Objectives: Despite substantial research on tRNA genes in various kingdoms, a comprehensive analysis of their conservation and the status of tandem duplication events throughout the tree of plant species studied has yet to be conducted. Methods: The tRNA genes from 50 plant species were identified, and gene length, intron length, and GC content were characterized. Then, identical, tandemly duplicated tRNA genes were analyzed according to the sequence identity and phylogenetic tree. Results: In this study, a total of 28,262 tRNA genes were identified across 50 plant species, encompassing eight divisions within the plant kingdom. tRNA gene length ranged from 62 to 98 bp and its abundance was found to have no correlation with genome size. The intron-containing tRNA genes are ubiquitously presented in all 50 plant species studied, and the most abundant were tRNA^Met_CAT^ and tRNA^Tyr_GTC^. A total of 578 identical tandemly duplicated tRNA gene pairs were identified and grouped into 410 clusters with 26 tRNA genes to the upmost. Different types of tandem duplication were identified as well, e.g., double-, triple-, and quintuple-tRNA genes, which were repeated for varied times. Tandemly located tRNA gene pairs with anticodons to proline were found to be widely spread in 33 plant species, including both lower and higher plants. Conclusions: The tRNA genes in different plants are highly conserved in terms of gene length, intron length, GC content, and sequence identity, with especially strong evidence for the strong sequence and structural conservation of tRNA genes, and the tandem duplication is an important driving for the tRNA gene evolution across diverse plant species.

## 1. Introduction

Transfer RNA (tRNA) plays a critical role in protein synthesis by bridging genetic code and corresponding amino acids and a central role in genetic code expansion [1]. tRNAs exhibit molecular hallmarks of ancient origin, functioning as living fossils that preserve primordial genetic coding mechanisms [2]. Beyond their canonical role in translation, tRNAs are reported to participate in tetrapyrrole biosynthesis, mRNA stabilization and transport, and serve as primers for viral RNA reverse transcription [3,4,5,6]. Additionally, tRNA-like structures are functionally significant components of RNA viral genomes. Notably, tRNA cleavage generates tRNA-derived RNAs (tDRs), which constitute an important class of regulatory small non-coding RNAs involved in gene expression control [7,8].

Transfer RNA (tRNA) molecules are typically defined by a common secondary structure consisting of one amino acid acceptor stem and four principal loops: the Dihydrouridine (D) loop, anticodon loop, TΨC loop, and variable loop. The size of the variable loop is the primary contributor to overall tRNA length heterogeneity. Substantial research has focused on identifying tRNA-coding genes across animals, plants, and microbes [9,10,11,12], with efforts centered on cataloging these genes and describing their basic characteristics, including gene and intron length. Despite this progress, a comprehensive understanding of tRNA gene evolution in plants is still lacking.

The organization of genomes is shaped by dynamic processes; besides genome duplication, there are at least three other modes, namely local (tandem) duplication, chromosomal segment duplication, and single-gene transposition–duplication [13,14,15,16]. Segmental duplications and large-scale rearrangements drive evolutionary innovation and adaptation [13,14]. Tandem duplication of tRNA genes serves as a fundamental evolutionary force, producing homologous tRNA clusters via localized genomic amplification [12,15]. In *Arabidopsis thaliana*, chromosome 1 harbors two prominent tRNA gene clusters resulting from extensive duplication events [17]. The first cluster comprises 27 tandemly duplicated tRNA^Pro^ genes, while the second consists of 27 consecutive tRNA^Tyr^–tRNA^Tyr^–tRNA^Ser^ repeat units. In *Zea mays*, a tandem repeat of 28 tRNA^Ile^ was found exclusively in chromosome 2 [12].

It is postulated that all tRNA genes (tDNAs) derive from an ancestral ‘proto-tRNA’ [18]. The evolutionary trajectory of genetic code is hypothesized to sector from a glycine code to 4 amino acid codes, then to 8 amino acid codes and then to 16 amino acid codes, and finally to the standard 20 amino acid codes with stops [19,20]. Previous research revealed divergent structural evolution patterns among *Arachnid* tRNA families, alongside evidence for parallel loss of tRNA arm-encoding sequences in an ancient, phylogenetically diverse animal clade [21]. However, the tRNA gene evolution pattern in plants has not been thoroughly investigated.

This study systematically annotated tRNA genes across 33 plant species, integrating analyses of structural features (length, intron length, GC content), sequence conservation, correlation among amino acid composition, codon usage bias and tRNA abundance, phylogenetic relationships, gene duplication patterns (including tandem arrays), and anticodon shifts. These multidimensional data revealed fundamental patterns of tRNA conservation and parallel evolution, providing new insights into plant tRNA gene evolution.

## 2. Materials and Methods

### 2.1. tRNA-Coding Gene Identification

Nuclear genome sequence, coding sequence, and protein sequence of 50 plant species were downloaded from phytozome (Supplementary Table S1). tRNA-coding genes were annotated by tRNAscan-SE (2.0.12) using “-H” and “-y” for eukaryotic tRNAs and then filtrated for high confidence sets using EukHighConfidenceFilter [12,22]. Minimum Fold Energy (MFE) of each tRNA gene was calculated by RNAFold [23]. The secondary structure of tRNA genes was displayed using VARNA GUI [24].

### 2.2. Sequence Alignment and Kn/Ks Estimation

Multiple sequence alignment of tRNA genes of identical-sequence, intron-containing tRNA genes was performed by multialin (http://multalin.toulouse.inra.fr/multalin/) (accessed on 15 October 2025). Global alignment of tRNA gene pairs was performed by Needle [25] and then sequence identity was obtained. Kn/Ks between tRNAs was calculated by KaKs_Calculator 3.0 [26]. tRNA gene sequence pairs were first compiled into *.fasta format using R, then aligned using clustalo and converted into phylip format through AXTConvertor to *.axt files compatible with KaKs_Calculator. Synonymous substitution rates were calculated using the default transition/transversion ratio (ω = 0.618).

### 2.3. GC Content Calculation

GC content of tRNA-coding genes was calculated by a window of 5 bp and step of 1 bp using R script, and the relative position of each window was normalized against the total length of each tRNA-coding gene. The fitting curve and confidence interval of the average GC content of each species were fitted by the method of ‘loess’ using ggplot2 v.4.0 package of R.

### 2.4. Phylogenetic Analysis of tRNA Genes

Fasta files of all tRNA gene sequences annotated above were formatted with R scripts. Then, a database of all tRNA genes was created by the build-in function of createdb from MMseqs2 (Many-against-Many sequence searching) [27]. Then, sequences were clustered with a minimum sequence identity of 0.9 and coverage of 0.8 (--min-seq-id 0.9 -c 0.8). The number of tRNA-coding genes with specific anticodons from different species was statistically analyzed and displayed in heatmap using ComplexHeatmap [28]. tRNA-coding genes with different anticodons were separated into different fasta files, and multiple sequence alignment was performed using clustalo, and then the best models for each tRNA-coding gene set were identified with BIC by the built-in model_finder in the IQ-TREE 2 [29]. After that, the phylogenetic tree was constructed using the best models inferred with bootstrap of 1000 times (Supplementary Table S2). The consensus tree was displayed by FigTree (v1.4.5_pre).

### 2.5. Identification of Tandem Duplication Event in tRNA Genes

Based on the annotation of tRNA genes across various plant species, tRNA gene pairs and clusters located on the same chromosome or scaffolded with a physical distance of less than 1 kb were initially identified and defined as tandem duplications. Additionally, for clusters composed of gene pairs with a sequence similarity below 100%, unique tRNA gene sequences were used for further screening. Clusters in which different combinations of tRNA genes recurred, and where tRNA genes sharing the same anticodon exhibited identical sequences, were also defined as tandem repeats.

## 3. Results

### 3.1. tRNA-Coding Genes in 50 Plant Species

To better understand tRNAs in plant species, we selected 50 plants, including eight divisions, namely Angiospermae (36), Bryophyta (4), Chlorophyta (4), Lycopodiophyta (2), Marchantiophyta (1), Pinophyta (1), Pteridophyta (1), and Rhodophyta (1), based on their phylogenetic positions in the plant kingdom (Supplementary Table S1 and Figure S1). Nuclear genome sequences of these plant species were obtained from Phytozome (Supplemental Table S1). A total of 28,262 high confident tRNA-coding genes were identified across all 50 plant species (Supplementary Table S3). The tRNA-coding gene length ranged from 62 to 98 bp and peaked at 72 bp and 82 bp (Figure S2A). The secondary structure of some representative tRNA-coding genes showed that the amino acid receptor arm was misformed in tRNA genes less than 70 bp, and longer tRNA genes usually possessed a bigger D-loop, T-loop, or variable loop (Figure S2B).

**Figure 1:**
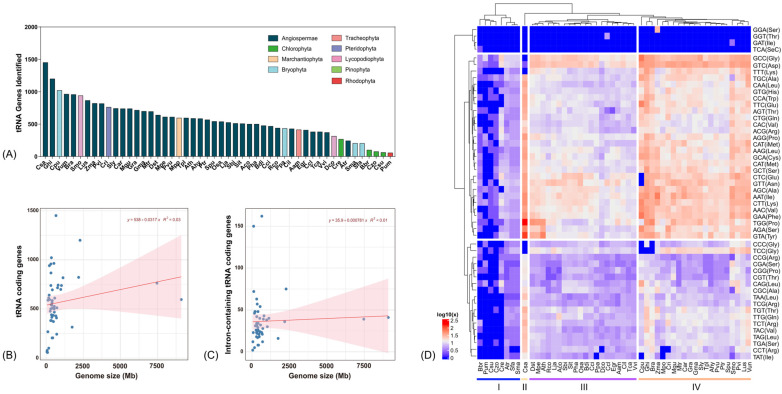
tRNA-coding genes identified in different plant species. (**A**) tRNA-coding genes identified in different species; (**B**) linear regression between genome size and total tRNA genes in all 50 plant species; (**C**) linear regression between genome size and intron containing tRNA genes in all 50 plant species; (**D**) heatmap of tRNA isoacceptor abundance in 50 plant species, clustered by Euclidean and complete linkage algorithms as default in ComplexHeatmap.

The total number of tRNA-coding genes varied among species, ranging from 56 in red algae (*Pum*) to 1451 in *Camelina sativa* (*Csa*) (Supplementary Table S4). Generally, tRNA-coding genes identified in species of Rhodopthyta (*Pum*) and Chloraphyta (*Bbr*, *Czo*, and *Csu*) showed the least abundance (≤100), and two Angiospermae species (*Csa* and *Ghi*) and one Bryophyta species (*Cpu*) identified more than 1000 tRNA-coding genes (Figure 1A). Correlation analysis revealed a weak positive but not significant relationship between tRNA-coding gene number and genome size (r = 0.18, *p* = 0.21), as well as between intron-containing tRNA-coding genes and genome size (r = 0.04, *p* = 0.77). This was further supported by linear regression between genome size and tRNA-coding genes, as well the intron-containing tRNA-coding genes (R^2^ = 0.03, and 0.01, respectively) (Figure 1B,C; Supplementary Figure S3 and Table S4).

Based on their anticodons, the tRNA genes were classified into 49 distinct types corresponding to 22 amino acids. This set includes the 20 standard amino acids, initiated methionine (iMet), and selenocysteine (SeC). tRNA^iMet_CAT^ was ubiquitously presented in all 50 plant species with 1–34 copies, while tRNA^SeC_TCA^ was only identified in four Chlorophytae species (Green algae, *Bbr*, *Cre*, *Csu*, and *Czo*) with extremely low abundance (one or two tRNA-coding genes in each species). The analysis revealed that 15 anticodons corresponding to 14 amino acids and a stop codon were absent across all 50 plant species. The coding gene for tRNA^Asp_GTC^ was the most numerous in 17 species in Agiospermae, as well as in Lycopodiophyta, Marchantiophyta, and Pinophyta. Conversly tRNA^Gly_GCC^ was the most abundant type in all four species belonging to Bryophyta division (Supplementary Table S5). Based on tRNA gene abundance, species were classified into four distinct clusters. Cluster I was primarily composed of Angiospermae, Chlorophyta, Rhodophyta, and Bryophyta, with algal species being exclusively found in this cluster. In contrast, Clusters III and IV exhibited a mixture of species from different taxonomic divisions (Figure 1D).

### 3.2. Intron-Containing tRNAs in Plants

**Figure 2:**
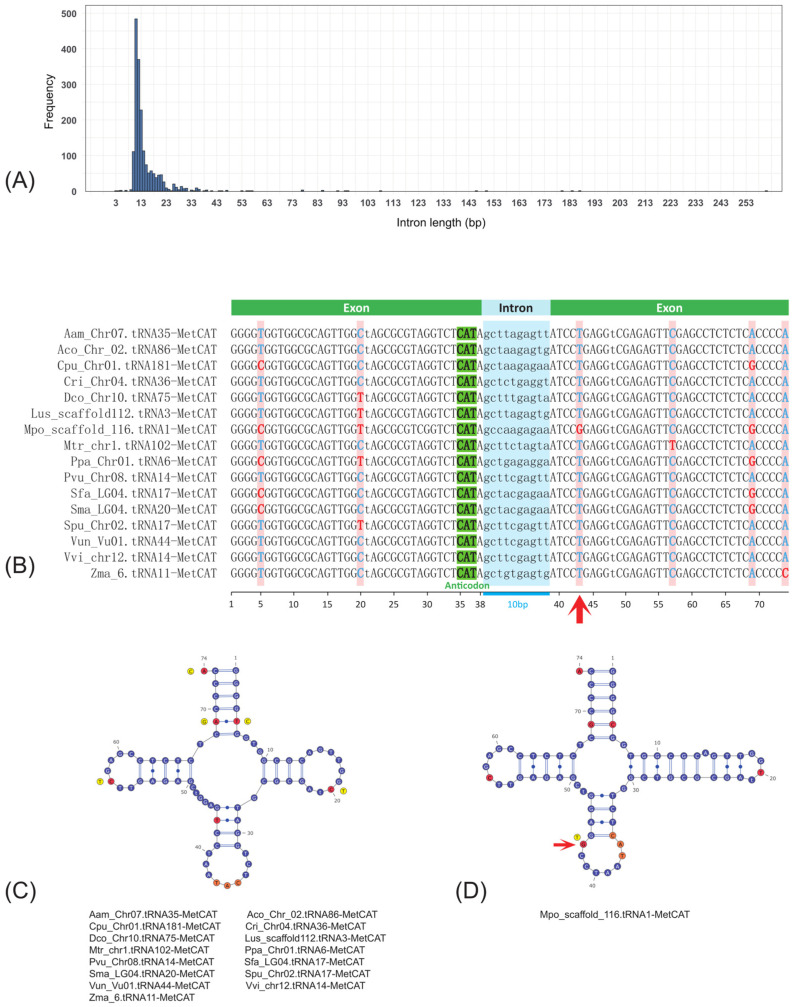
Conservation of tRNAMet_CAT-coding genes among different species. (**A**) Distribution of intron length among 1826 intron-containing tRNA genes; (**B**) multiple sequence alignment of tRNA^Met_CAT^-coding genes; (**C**) overlap of secondary structure of tRNA^Met_CAT^ from 15 different species; (**D**) secondary structure of tRNA^Met_CAT^ from *Mpo.* Red arrow indicates the base variation in the anticodon arm resulting in significant secondary structure change.

Among the 50 plant species, 1826 intron-containing tRNA genes were identified (Supplementary Table S3). The ratio of intron-containing tRNA genes to total tRNA-coding genes (I/T) ranged from 3.23% (*Csu*) to 56.18% (*Cre*), with 88.00% of those species less than 10.00%. Of the six species with I/T ratio larger than 10%, the top three were algae (*Cre*, *Pum*, and *Czo*), and the others were eudicots (*Ath*, *Cci*, and *Csa*) (Supplementary Table S4). The intron length of tRNA genes ranged from 3 bp to 261 bp, and peaked at 12 bp, with an average of 15.15 bp (Figure 2A). The 93.21% of tRNA genes possessed an intron length of less than 22 bp. These intron-containing tRNA genes covered all 20 standard amino acids, but 86.87% were associated with Met (methionine) and Tyr (tyrosine), with 36.91% and 49.95% for Met and Tyr, respectively. All tRNA^Met^- and tRNA^Tyr^- coding genes (100.00%) had the anticodons of CAT and GTA, respectively (Supplementary Table S6). Both tRNA^Met_CAT^ and tRNA^Tyr_GTA^ isotypes presented in 48 plant species studied with the exception of *Pum* and *Csu* for tRNA^Met_CAT^ and *Pum* and *Bbr* for tRNA^Tyr_GTA^, all of which belonged to algae. In addition, other algae (*Czo*, and *Cre*) species also showed low abundance (≤9). Take tRNA^Met_CAT^ as an example; tRNA genes with an intron length of 10 bp from different species showed six variation loci, of which two were in the loop, three were on the stem, and one was on the acceptor stem. Most of those variation loci do not influence the secondary structure except for the T43G on the anticodon stem, which resulted in a significant structure change in *Mpo* (Figure 2B,C).

### 3.3. The GC Content of tRNA-Coding Genes

**Figure 3:**
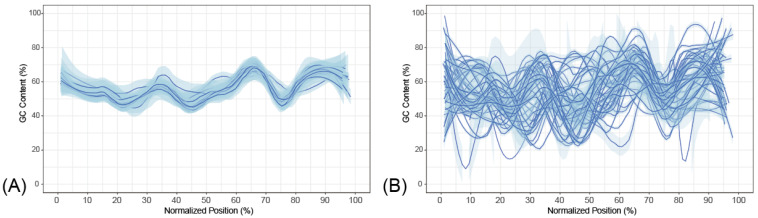
GC content fitting curve of tRNA-coding genes in different plant species. GC content of tRNA isodecoder genes classified by species (**A**) and anticodon (**B**). The x-axis represents relative position of all tRNA-coding genes.

Given the structural conservation of tRNAs, which share common arms and loops, their coding sequences were expected to exhibit evolutionary conservation. In this study, the total genomic GC content across 50 plant species ranged from 27.94% to 65.68%. Monocots displayed relatively higher GC content (38–47%) compared to eudicots (27–39%), while algae exhibited the highest values (>47%) (Supplementary Table S4). In contrast, the average GC content of tRNA genes across all species was 56.72% and ranged from 38.36 to 71.95% (Supplementary Table S3). However, the average GC content of tRNA genes with different anticodons showed greater variability (48.61–63.32%) compared to that of different species which ranged from 55.33 to 58.20% (Supplementary Table S3). Furthermore, the position-dependent GC content of tRNA-coding sequences followed a consistent trend across all 50 species, as demonstrated by the GC content fitting curve (Figure 3). Specifically, higher GC content was observed in regions corresponding to tRNA stem structures (0.00–5.00%, 60.00–70.00%, and 80.00–100.00% of the gene length). The anticodon region (located at 45.37–48.03% of the total length) exhibited the lowest GC content across the entire tRNA sequence (Figure 3A). However, when the GC content was fitted by anticodon across different species, the GC content trend along tRNA-coding gene position varied a lot (Figure 3B); this might be explained by the significant variation in the whole genome GC content.

### 3.4. Phylogenetic Analysis of tRNA-Coding Genes

**Figure 4:**
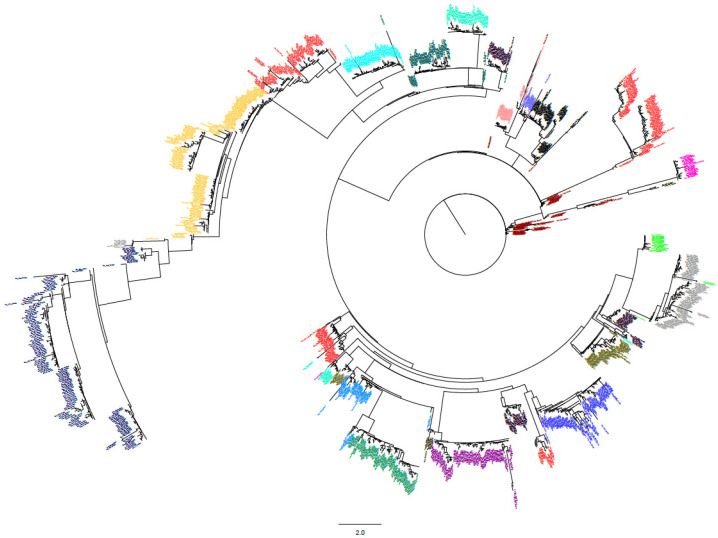
Phylogenetic tree of 6580 unique tRNA gene sequences. tRNA genes of different anticodon were indicated by different colors.

To elucidate the evolutionary patterns of tRNA genes in plant species, 28,262 tRNA-coding sequences were classified into eight clusters (zero to seven) based on sequence similarity and coverage. A phylogenetic tree was constructed for each cluster (Supplementary Figure S2). Within each cluster, tRNA genes carrying specific anticodons exhibited enrichment patterns (Table 1). Notably, tRNA genes targeting the same amino acid were distributed across distinct clusters. For example, tRNA^Gly^ genes with GCC, CCC, and TCC anticodons were predominantly enriched in Clusters 0, 1, and 2, respectively (Table 1; Supplementary Figure S3). Based on the sequence identity, a total of 6580 unique tRNA gene sequences were identified, and then each tRNA gene was assigned with a unique tRNA name based on the sequence (Supplemental Table S3). Phylogenetic tree of unique tRNA genes showed that the tRNA isotypes of same anticodon tended to group together but with few exceptions, suggesting profound conservation within tRNA gene isotypes (Figure 4).

### 3.5. Identification of Tandem Duplication Event During tRNA Gene Expansion

Among all 28,262 tRNA genes, 2720 pairs were found within a physical distance of less than 1 kb. Among these, 578 pairs (21.25% of the total) exhibited 100% identical sequences. These identical tRNA gene pairs were clustered into 410 contiguous intervals, corresponding to 175 unique tRNA gene sequences distributed across 39 different species (Supplementary Table S7). These species belong to seven distinct taxonomic groups, with Angiospermae comprising the majority. Within those identical tRNA genes, a total of 43 distinct anticodons corresponding to 20 standard amino acids and iMet were identified, of which Pro (tRNA^Pro_(T/A/C)GG^) ranked the first with a total of 100 tandem clusters in 27 species (Supplementary Table S7). Among the tandem repeats of identical tRNA sequences, only a few species of Bryophytes (*Cpu* and *Ppa*) and algae (*Cre*) were involved. Interestingly, a total of 58 identical tRNA genes with isotypes of tRNA^Ile_AAT^ and tRNA^Cys_GCA^ were identified to be tandemly located and grouped into three clusters (each contained 26, 18, and 14 tRNA genes, respectively) in *Cpu*.

**Figure 5:**
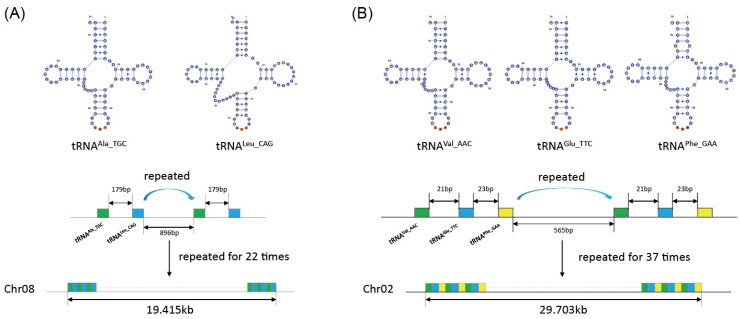
Tandem duplication of tRNA genes in *Cpu*. (**A**) Tandem duplication of tRNA^Ala_TGC^ and tRNA^Leu_CAG^ on Chr08 in *Cpu*; (**B**) tandem duplication of tRNA^Val_AAC^, tRNA^Glu_TTC^, and tRNA^Phe_GAA^ on Chr02 in *Cpu.* Duplication of same tRNA isotypes showed sequence identity of 100%.

For tandemly repeated tRNAs with sequence divergence, 2142 pairs showed varying degrees of sequence similarity from 22.8% to 98.9% (Supplementary Table S8). Among these tRNA gene pairs, sequence similarity above 74.4% consistently targeted the same amino acid, and 77.41% of which showed no changes in anticodon. These 2142 tRNA gene pairs were then grouped into a total of 1338 tRNA gene clusters based on physical distance within 1 kb between adjacent tRNA genes, within which the tRNA gene number of each cluster ranged from two to 110. Notably, ten clusters from *Ath*, *Cre*, and *Cpu* showed two to five different tRNA isotypes repeatedly arranged, suggesting that those clusters were of different types of tandem duplication (Figure 5; Supplementary Table S9).

### 3.6. Conservation of Proline tRNA Isotypes Among Different Plant Species

During tandem duplication event identification, 77.41% of tandemly located tRNA genes showed the same anticodon, the rest of which were targeted to the same amino acid but with degenerated anticodons. Among the tandemly located tRNA gene pairs, 220 pairs were found to target the same amino acid but with different codons. These involved Ala, Gln, Leu, Ser, Thr, Pro, and Val. Of these, 166 pairs were related to proline and were distributed across 33 different species, including some lower plants such as *Cre*, *Cpu*, *Ppa*, and *Tpl*. Sequence analysis revealed that the sequence identity of these tRNA gene pairs was consistently greater than 79%. For pairs with similarity exceeding 90%, the maximum number of base variations was six, while the minimum was only a single base substitution. These variations have a minimal impact on the secondary structure of the tRNA, and compensatory mutations were observed in the base-paired residues located within the stem regions. Kn/Ks values between those tRNA pairs were all less than 0.40, suggesting a recent origination both in the lower and higher plant species (Supplementary Table S8).

## 4. Discussion

### 4.1. Conservation of tRNA Genes Among Plant Species

In this study, a total of 28,262 tRNA genes across 50 plant species were identified with high confidence and conserved secondary structure (Figure 1B; Supplementary Table S3). By checking the tRNA gene number in species reported by previous studies, e.g., PlantRNA database, tRNA genes identified in this study showed highly repeatability [12,30]. A weak and nonsignificant positive correlation between tRNA gene number and genome size was observed in this study (Figure 1A,B; Supplementary Figure S4), which contradicts previous findings; this is probably due to the inclusion of some species of a large genome size, e.g., *Zma*, *Ghi*, *Cri* and *Tpl*. The evolutionary conservation of tRNA genes, well-documented in previous studies [10,12], is further corroborated by the findings of the present investigation. In this study, intron-containing tRNA genes were identified across all 50 examined plant species (Supplemental Table S3). Of those intron-containing tRNAs, intron length is more conserved within species than between species, with an exception of tRNA^Met_CAT^ and tRNA^Tyr_GTA^, whose intron length is conserved between species, suggesting that both of them are of ancient origin (Supplemental Table S3). In terms of GC content, the tRNA genes is more conserved in different species than of different anticodons (Figure 3). We also noticed that the tRNA^pro^ genes were tandemly located in 33 species, including both lower plants and higher plants (Supplementary Table S3), suggesting that the tRNA^pro^ is of ancient origin and conserved among different species, which possibly supports the opinion that proline might be the first amino acid in the primitive genetic code [31]. Collectively, these findings provide robust evidence for the evolutionary conservation of tRNA genes across different plant species.

### 4.2. Tandem Duplication Is a Universal Driving Force for the tRNA Gene Evolution

In this study, a total of 578 tRNA gene pairs were identified to be tandemly duplicated within 1 Kb and have a sequence identity of 100%, and were then grouped into 410 distinct gene clusters. For the 2142 nonidentical but tandemly located tRNA gene pairs, a total of 1338 gene clusters were obtained. Different types of tandem duplication, e.g., single-, double-, triple-, and quintuple-tRNA genes, were also identified in different plant species (Figure 5; Supplementary Tables S8 and S9). Those tRNA genes showed a broad coverage in terms of species (39), amino acids (21), and anticodon (43). Tandem duplication is a common driving force in numerous genome expansion. In bacteria, large-scale tandem duplication is supposed to be an important way in the birth of bacterial tRNA genes [16]. Monloy et al. reported tandem tRNA gene clusters in *Ath* and *Zea* [12,16]. In our study, those regions were also identified in this study but with varied repeated times; this is probably because of the stricter standard used in this study. It is noteworthy that a bryophyte, *Cpu*, exhibits a particularly widespread distribution of tandem tRNA repeats. This is characterized by three distinct genomic intervals comprising large clusters of 26, 18, and 14 identical tRNA genes, alongside five additional intervals where tandem repeats of 2 and 3 tRNA genes repeated for as much as 37 times (Figure 5). Besides *Cpu*, another lower plant, algae (*Cre*), was also found to be involved in gene clusters, indicating that tandem duplication widely exists in plants as well as archaea and higher primates [9,32].

## 5. Conclusions

This study resolves a key gap in our understanding of plant tRNA gene evolution. By conducting a large-scale comparative genomic analysis of 28,262 tRNA genes covering 49 anticodons across 50 plant species. The length of tRNA genes ranged from 62 bp to 98 bp, and only 1826 tRNA genes contained intron, which ranged from 3 bp to 261 bp and peaked at 12 bp. Intron-containing tRNA genes covered all 50 plant species, and most of those tRNAs were related to tRNA^Met_CAT^ and tRNA^Tyr_GTA^. GC content of tRNA genes showed a similar trend in different species but are diverse in tRNA genes of different anticodons. Crucially, the pervasive tandem duplication of tRNA genes were identified across 39 different plant species and played significant role in driving the expansion and functional diversification of tRNA isotypes.

## Figures and Tables

**Table 1:** Enriched tRNA genes in different clusters classified by anticodon.

Clusters	Enriched tRNAs
0	GCA(Cys); CGA(Ser); CAA(Leu); CCT(Arg); CAT(Met); GCC(Gly); GTT(Asn)
1	AGT(Thr); CTC(Glu); CTG(Gln); CCC(Gly); CCA(Trp); TTT(Lys)
2	TCC(Gly); CTT(Lys); AAC(Val); CAC(Val); AAT(Ile); GTC(Asp); CAG(Leu); TAA(Leu); TAT(Ile)
3	TCT(Arg); CGT(Thr); GCT(Ser); AGG(Pro); TGG(Pro)
4	AGA(Ser); TTC(Glu)
5	GAA(Phe); AGC(Ala); GTG(His); CGC(Ala); TAG(Leu)
6	CCG(Arg); TCG(Arg); TTG(Gln); AAG(Leu); TGT(Thr)
7	TGC(Ala); ACG(Arg); TGA(Ser); TAC(Val)

## Data Availability

The original contributions presented in this study are included in the article/Supplementary Material. Further inquiries can be directed to the corresponding authors.

## Supplementary Materials

The following supporting information can be downloaded at https://www.mdpi.com/article/10.3390/genes16111307/s1, Supplementary Figure S1. Phylogenetic tree of 50 plant species according to phytozome. Supplementary Figure S2. Gene length and representative secondary structure of tRNA genes identified in 50 plant species. Supplementary Figure S3. Heatmap of tRNA genes with different anticodon in different clusters. Supplementary Table S1. Genomic data resource collection in this study. Supplementary Table S2. Best model for phylogenetic tree construction in different clusters. Supplementary Table S3. tRNA-coding genes identified by tRNAscan-SE in 50 plant species. Supplementary Table S4. Basic statistics of tRNA-coding genes. Supplementary Table S5. Most abundant tRNA-coding genes in different plant divisions. Supplementary Table S6. Intron-containing tRNA-coding genes. Supplementary Table S7. Tandemly duplicated tRNA gene clusters. Supplementary Table S8. Nonidentical and tandemly located tRNA gene pairs. Supplementary Table S9. Tandem duplication events in different species.

## References

[1] Reynolds N.M., Vargas-Rodriguez O., Söll D., Crnković A. (2017). The central role of tRNA in genetic code expansion. Biochim. Biophys. Acta (BBA)-Gen. Subj..

[2] Shimizu N. (1971). Studies on nucleic acids of living fossils. J. Biochem..

[3] Agrawal S., Karcher D., Ruf S., Bock R. (2020). The functions of chloroplast glutamyl-tRNA in translation and tetrapyrrole biosynthesis. Plant Physiol..

[4] Liu B., Cao J., Wang X., Guo C., Liu Y., Wang T. (2021). Deciphering the tRNA-derived small RNAs: Origin, development, and future. Cell Death Dis..

[5] Wilusz J.E. (2015). Controlling translation via modulation of tRNA levels. Wiley Interdiscip. Rev. RNA.

[6] Zhang W., Thieme C.J., Kollwig G., Apelt F., Yang L., Winter N., Andresen N., Walther D., Kragler F. (2016). tRNA-related sequences trigger systemic mRNA transport in plants. Plant Cell.

[7] Li Y., Zhou H. (2009). tRNAs as regulators in gene expression. Sci. China Ser. C Life Sci..

[8] Chery M., Drouard L. (2023). Plant tRNA functions beyond their major role in translation. J. Exp. Bot..

[9] Bermudez-Santana C., Attolini C.S.O., Kirsten T., Engelhardt J., Prohaska S.J., Steigele S., Stadler P.F. (2010). Genomic organization of eukaryotic tRNAs. BMC Genom..

[10] Michaud M., Cognat V., Duchêne A.M., Maréchal-Drouard L. (2011). A global picture of tRNA genes in plant genomes. Plant J..

[11] Shepherd J., Ibba M. (2015). Bacterial transfer RNAs. FEMS Microbiol. Rev..

[12] Monloy K.C., Planta J. (2024). tRNA gene content, structure, and organization in the flowering plant lineage. Front. Plant Sci..

[13] Lynch M., Walsh B. (2007). The Origins of Genome Architecture.

[14] Tang H. (2007). Genome assembly, rearrangement, and repeats. Chem. Rev..

[15] Freeling M. (2009). Bias in plant gene content following different sorts of duplication: Tandem, whole-genome, segmental, or by transposition. Annu. Rev. Plant Biol..

[16] Ayan G.B., Park H.J., Gallie J. (2020). The birth of a bacterial tRNA gene by large-scale, tandem duplication events. Elife.

[17] Theologis A., Ecker J.R., Palm C.J., Federspiel N.A., Kaul S., White O., Alonso J., Altafi H., Araujo R., Bowman C.L. (2020). Sequence and analysis of chromosome 1 of the plant *Arabidopsis thaliana*. Nature.

[18] Eigen M., Lindemann B.F., Tietze M., Winkler-Oswatitsch R., Dress A., Von Haeseler A. (1989). How old is the genetic code? Statistical geometry of tRNA provides an answer. Science.

[19] Kim Y., Opron K., Burton Z.F. (2019). A tRNA-and anticodon-centric view of the evolution of aminoacyl-tRNA synthetases, tRNAomes, and the genetic code. Life.

[20] Lei L., Burton Z.F. (2020). Evolution of life on Earth: tRNA, aminoacyl-tRNA synthetases and the genetic code. Life.

[21] Masta S.E., Boore J.L. (2008). Parallel evolution of truncated transfer RNA genes in Arachnid mitochondrial genomes. Mol. Biol. Evol..

[22] Chan P.P., Lin B.Y., Mak A.J., Lowe T.M. (2021). tRNAscan-SE 2.0: Improved detection and functional classification of transfer RNA genes. Nucleic Acids Res..

[23] Lorenz R., Bernhart S.H., Höner zu Siederdissen C., Tafer H., Flamm C., Stadler P.F., Hofacker I.L. (2011). ViennaRNA Package 2.0. Algorithms Mol. Biol..

[24] Darty K., Denise A., Ponty Y. (2009). VARNA: Interactive drawing and editing of the RNA secondary structure. Bioinformatics.

[25] Rice P., Longden I., Bleasby A. (2000). EMBOSS: The European molecular biology open software suite. Trends Genet..

[26] Zhang Z. (2022). KaKs_Calculator 3.0: Calculating selective pressure on coding and non-coding sequences. Genom. Proteom. Bioinform..

[27] Steinegger M., Söding J. (2017). MMseqs2 enables sensitive protein sequence searching for the analysis of massive data sets. Nat. Biotechnol..

[28] Gu Z. (2022). Complex heatmap visualization. Imeta.

[29] Minh B.Q., Schmidt H.A., Chernomor O., Schrempf D., Woodhams M.D., Von Haeseler A., Lanfear R. (2020). IQ-TREE 2: New models and efficient methods for phylogenetic inference in the genomic era. Mol. Biol. Evol..

[30] Cognat V., Pawlak G., Pflieger D., Drouard L. (2022). PlantRNA 2.0: An updated database dedicated to tRNAs of photosynthetic eukaryotes. Plant J..

[31] Komatsu R., Sawada R., Umehara T., Tamura K. (2014). Proline might have been the first amino acid in the primitive genetic code. J. Mol. Evol..

[32] Morgado S.M., Vicente A.C.P. (2019). Exploring tRNA gene cluster in archaea. Mem. Inst. Oswaldo Cruz.

