# Supplementary material for: Conservation and Tandem Duplication of tRNA Genes in Plant Species
Source: Genes (Basel). 2025 Nov 1;16(11):1307. doi: 10.3390/genes16111307 (PMC12652284; doi:10.3390/genes16111307)
Supplement: Supplementary file 1 [file genes-16-01307-s001.zip › Figure S1.pdf]

Archaeplastida

- **Rhodophyta**
  - *Porphyra umbilicalis* (***Pum***)
- **Viridiplantae**
  - **Chlorophyte**
    - *Botryococcus braunii* (***Bbr***)
    - *Chlamydomonas reinhardtii* (***Cre***)
    - *Chromochloris zofingiensis* (***Czo***)
    - *Coccomyxa subellipsoidea* (***Csu***)
  - **Embryophyte**
    - *Ceratodon purpureus* (***Cpu***)
    - *Marchantia polymorpha* (***Mpo***)
    - *Physcomitrium patens* (***Ppa***)
    - *Sphagnum fallax* (***Sfa***)
    - *Sphagnum magellanicum* (***Sma***)
    - **Tracheophyte**
      - *Ceratopteris richardii* (***Cri***)
      - *Diphasiastrum complanatum* (***Dco***)
      - *Selaginella moellendorffii* (***Smo***)
      - *Thuja plicata* (***Tpl***)
      - **Angiosperm**
        - *Amborella trichopoda* (***Atr***)
        - **Eudicot**
          - *Aquilegia coerulea* (***Aco***)
          - **Pentapetalae**
            - *Amaranthus hypochondriacus* (***Ahy***)
            - **Asterids**
              - *Coffea arabica* (***Car***)
              - *Mimulus guttatus* (***Mgu***)
              - *Solanum lycopersicum* (***Sly***)
            - **Rosid**
              - *Eucalyptus grandis* (***Egr***)
              - *Vitis vinifera* (***Vvi***)
              - **Fabidae**
                - *Glycine max* (***Gma***)
                - *Lotus japonicus* (***Lja***)
                - *Medicago truncatula* (***Mtr***)
                - *Phaseolus vulgaris* (***Pvu***)
                - **Carya illinoinensis**
                  - *Carya illinoinensis* (***Cil***)
                - **Vigna unguiculata**
                  - *Vigna unguiculate* (***Vun***)
              - **Malvidae**
                - *Corymbia citriodora* (***Cci***)
                - **Malpighiales**
                  - *Linum usitatissimum* (***Lus***)
                  - *Salix purpurea* (***Spu***)
                  - *Ricinus communis* (***Rco***)
                - **SBM**
                  - **Brassicales-Malvales**
                    - *Theobroma cacao* (***Tca***)
                    - **Brassiceae**
                      - *Arabidopsis thaliana* (***Ath***)
                      - *Diptychocarpus strictus* (***Dst***)
                      - **Brassiceae**
                        - *Brassica rapa* (***Bra***)
                      - **Camelina**
                        - *Camelina sativa* (***Csa***)
                      - **Isatideae**
                        - *Myagrum perfoliatum* (***Mpe***)
                    - **Cotton**
                      - *Gossypium hirsutum* (***Ghi***)
                      - *Gossypium raimondii* (***Gra***)
                  - **Citrus**
                    - *Citrus clementina* (***Ccl***)
                    - *Poncirus trifoliata* (***Ptr***)
        - **Monocot**
          - *Acorus americanus* (***Aam***)
          - **Grass**
            - *Oryza sativa* (***Osa***)
            - **Brachypodium**
              - **Bdistachyon**
                - *Brachypodium distachyon* (***Bdi***)
              - **Panicoideae**
                - *Panicum virgatum* (***Pvi***)
                - *Setaria italica* (***Sit***)
                - **Maize**
                  - *Zea mays* (***Zma***)
                - **Phallii**
                  - *Panicum hallii* (***Pha***)
                - **Sorghum bicolor**
                  - *Sorghum bicolor* (***Sbi***)
