# Supplementary figures and images for: Conservation and Tandem Duplication of tRNA Genes in Plant Species
Source: Genes (Basel). 2025 Nov 1;16(11):1307. doi: 10.3390/genes16111307 (PMC12652284; doi:10.3390/genes16111307)

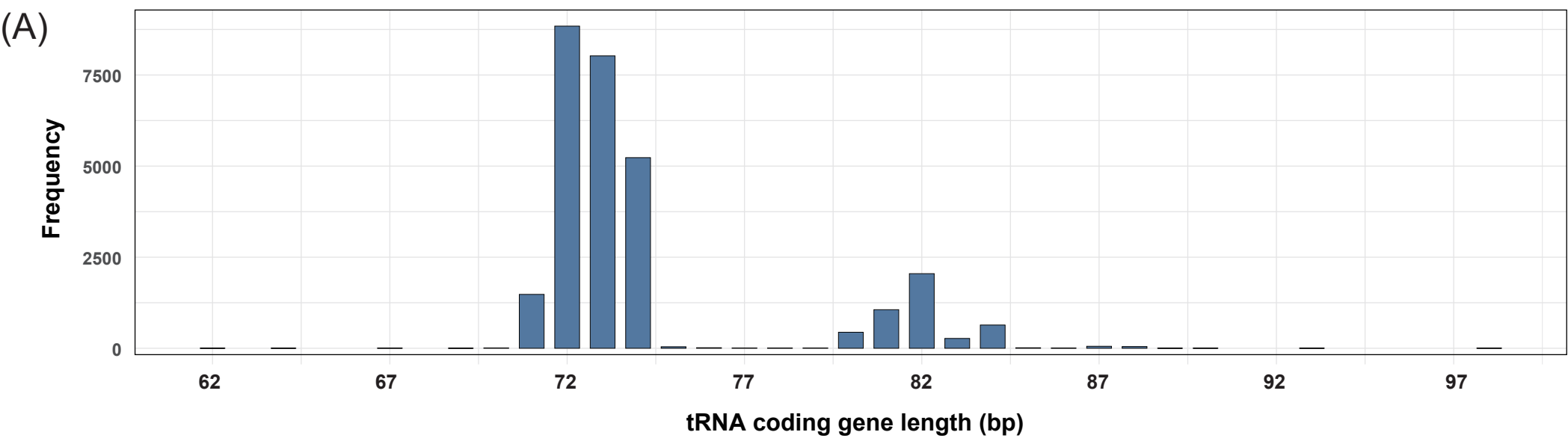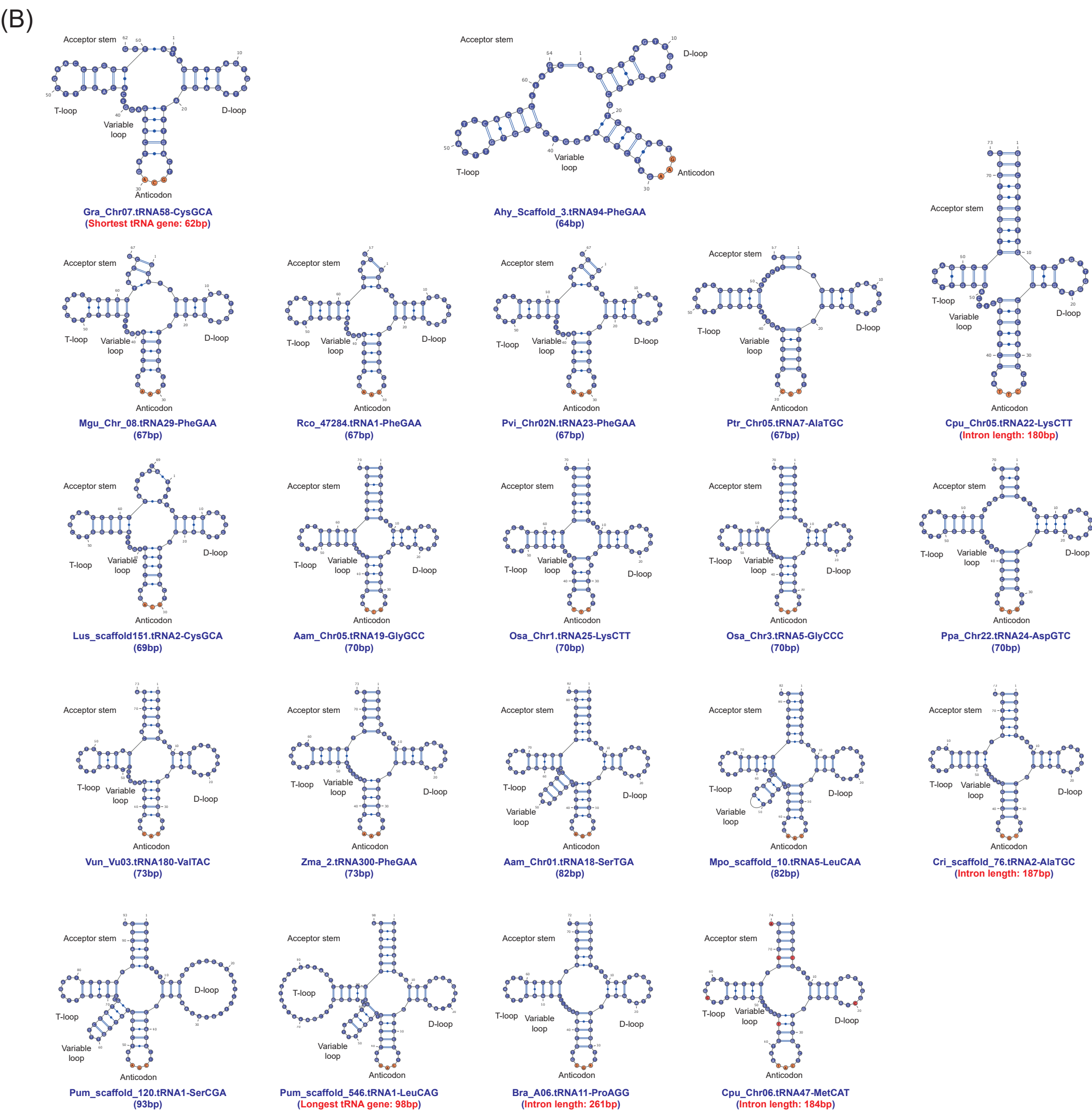

Supplement: Supplementary file 1 [file genes-16-01307-s001.zip › Figure S2.pdf]

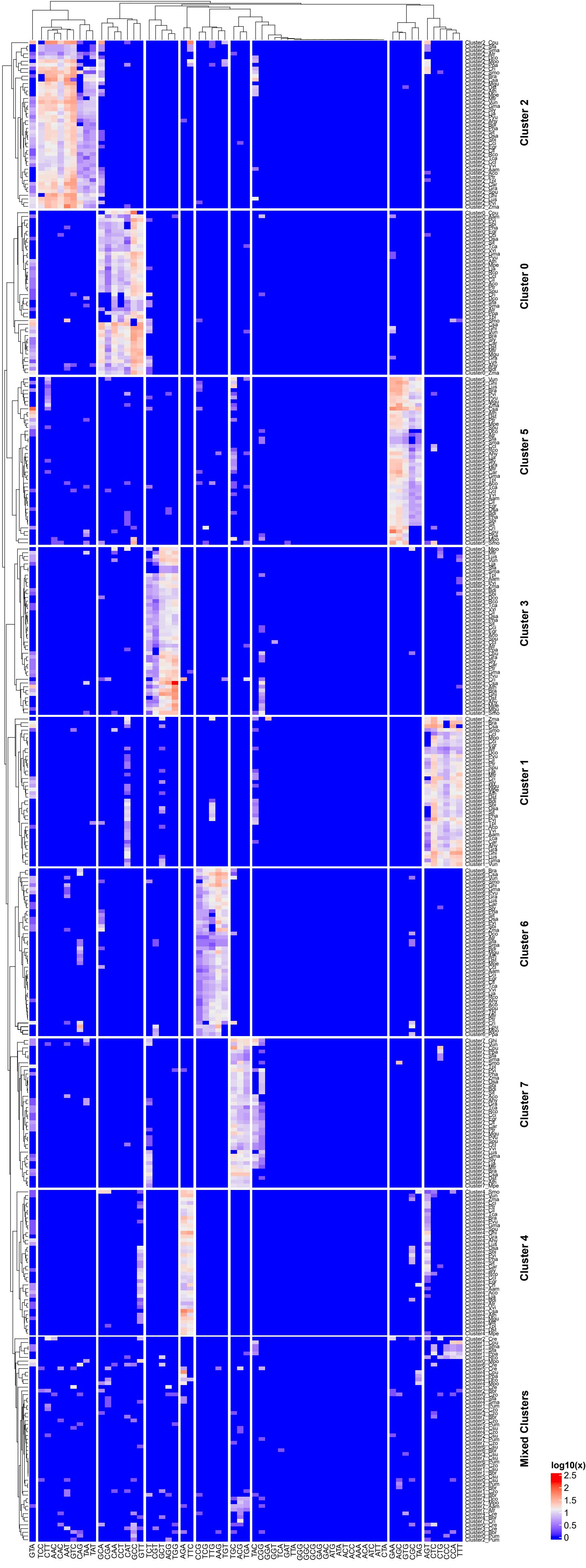

Supplement: Supplementary file 1 [file genes-16-01307-s001.zip › Figure S3.pdf]
